# Functional biology in its natural context: A search for emergent simplicity

**DOI:** 10.7554/eLife.67646

**Published:** 2021-06-07

**Authors:** Joy Bergelson, Martin Kreitman, Dmitri A Petrov, Alvaro Sanchez, Mikhail Tikhonov

**Affiliations:** 1 Department of Ecology & Evolution, University of ChicagoChicagoUnited States; 2 Department of Biology, Stanford UniversityStanfordUnited States; 3 Department of Ecology & Evolutionary Biology, Yale UniversityNew HavenUnited States; 4 Department of Physics, Washington University in St LouisSt. LouisUnited States; Université LavalCanada; Pennsylvania State UniversityUnited States

**Keywords:** eco-evolutionary dynamics, functional biology, ecological context, systems biology

## Abstract

The immeasurable complexity at every level of biological organization creates a daunting task for understanding biological function. Here, we highlight the risks of stripping it away at the outset and discuss a possible path toward arriving at emergent simplicity of understanding while still embracing the ever-changing complexity of biotic interactions that we see in nature.

## Introduction

*The problem is to construct a third view, one that sees the entire world neither as an indissoluble whole nor with the equally incorrect, but currently dominant, view that at every level the world is made up of bits and pieces that can be isolated and that have properties that can be studied in isolation. Both ideologies [...] prevent a rich understanding of nature and prevent us from solving the problems to which science is supposed to apply itself. *

*- *Lewontin, “Biology as Ideology”

*Almost without exception, when nuance is mentioned it is because someone is asking for more of it. I argue that [...] demanding more nuance typically obstructs the development of theory that is intellectually interesting, empirically generative, or practically successful.*

- Kieran Healy, “Fuck Nuance”

In the study of biology, we seek simplicity to foster understanding. This quest is driven not only by philosophical desire, but also empirical necessity. Nature brings us immeasurable complexity at every conceivable level of organization — ecological communities are composed of interacting networks of dynamically evolving players; populations of species are defined by membership in closely interacting groups competing for shared resources; cell behavior is governed by a myriad of overlapping and intertwined biochemical pathways and cell-cell interactions; gene regulation is carried out by a web of interacting transcription factors, modified by the cellular environment and epigenetic landscape. All are complex interwoven products of evolution, subject to continual change not by a grand designer but by tinkering and chance. To gain insights into this complexity, our solution as biologists has been to simplify, and this has led to great successes. But, as we will argue below, it has also come with a cost.

The first key level of simplification has been to separate function from evolutionary history, and to organize the study of biology into these two distinct pursuits: ‘How do things work?’ and ‘How did they get that way?’. In the pursuit of functional understanding, the next step has been to simplify the system by isolating the organism from its environmental context. The tool of the trade is a lab-bred model organism — reared on defined growth media, isolated from its natural biotic and abiotic environments, stripped of its natural genetic heterozygosity, and removed from daily and seasonal fluctuations. Humanized mouse models — specialized pure-breeding lines carrying human genes, cells, tissues, or organs — serve as a canonical example of the quest to reduce the complexity of a biological system by isolating its individual components.

Simplification has, likewise, been a key to success in evolutionary biology. It is achieved in a different way, however, by separating the study of processes acting on populations over ecological timescales from the consequences of these processes as revealed on phylogenetic timescales. This separation of timescales has been justified by the belief that ecological change can be rapid — wildfires are an extreme example — whereas evolutionary change in response to an ecological challenge, such as increasingly frequent occurrence of wildfire, is a slow and gradual process. As such, the study of evolution has primarily concerned itself with the inference of history. This principle operates even in population genetics, which in theory bridges ecological processes that govern the fitness of individuals, with evolutionary changes in phenotypes of species driven by adaptation. Most of its machinery aims at making inferences about ostensibly slow evolutionary processes from time-frozen snapshots of genetic variation, with the ecological processes being of relevance only in their long-term average behavior. In ecology, this separation of timescales allows the projection of dynamics from the measurement of contemporary interactions, a tactic that sets aside evolutionary processes. The perceived difference in timescales over which ecological and evolutionary processes act has led to the de facto intellectual divorce of the two — their apparent marriage in the many academic departments entitled ‘Ecology and Evolution’ persisting primarily in name alone.

We contend that while powerful, this current modality of experimental simplification — to a single lab strain in a single environment, or to a single species, or to a static timeframe — handicaps our ability to comprehend nature as it actually exists. Biological entities do not exist in isolation but rather have evolved, and continue to evolve, while enmeshed in a network of ever-changing biotic and abiotic interactions. Even our own human body is, in fact, an entire ecosystem of interacting species and evolving somatic lineages (Ley et al., 2006; Martincorena, 2019). As each of these multiple entities evolves, so too does the network of their interactions (e.g. Fiegna et al., 2015; Urban and Skelly, 2006). Function in these systems is intimately linked to eco-evolutionary dynamics and the eco-evolutionary dynamics are, of course, intimately linked to function. There is no separation of timescales, just as there is no separation of the fields of inquiry. It is all one thing, not merely as a philosophical stance but as an entirely practical matter.

While we fully embrace the goal of *simplicity of understanding*, we argue that this simplicity must come from a source other than system simplification and isolation at the initiation of inquiry. Indeed, stripping experimental systems from their essential eco-evolutionary context not only makes the resulting functional insights less relevant, it can make them positively misleading. We argue that this approach runs the risk of simplifying away the very phenomena we strive to understand; in essence, eliminating the subject of inquiry before the inquiry even begins.

Our aim here is not to engage in an age-old debate between reductionism and holism, nor argue for more context and nuance for their own sake. Instead, we argue that specifically now, with the recent advances in high-throughput measurement and high-performance computing, we might finally be in a position to interrogate biological systems in their fuller eco-evolutionary context, with the goal of exposing the hidden regularities and arrive at an *emergent* simplicity of understanding. It is essential to embrace this challenge head-on if we are to understand biological function in the appropriate and relevant context, which we furthermore argue is necessary for any practical application.

## Does it matter?

We have suggested that failure to consider eco-evolutionary context can lead us astray in the understanding of biological function. Is there any evidence that it in fact does? We believe that the answer is yes at multiple levels, from individual genes to complex communities of organisms. Indeed, even a cursory survey provides multiple compelling examples. At the lowest level of biological organization, the function of a single gene cannot be understood without regard to environmental context. The fact that approximately 35% of genes in *E. coli*, perhaps the best studied organism in the lab, have no known function (Ghatak et al., 2019) is certainly not due to lack of effort, but speaks to the difficulty of understanding organisms in isolation. The function of genes that are recalcitrant to mutational knockout analysis in the laboratory can be revealed under their natural biotic conditions (Barbaric et al., 2007; Chanin et al., 2019; Cruz et al., 2016; Hutchison et al., 2016; Lin et al., 2020; Richter et al., 2009; Ruff et al., 2015). It stands to reason that the full elucidation of gene function, regulatory architecture and developmental robustness will require investigation in an organism’s natural environment.

Similarly, the genetic architecture of phenotypic variation cannot be easily understood outside of the appropriate environmental context. For example, the key developmental genes underlying flowering in *Arabidopsis thaliana* have been carefully dissected in greenhouse and growth chamber experiments. However, when this same species is grown in natural conditions, GWAS experiments reveal that natural variation in flowering is in fact largely due to variation in circadian clock genes (Brachi et al., 2010). It should therefore not be surprising that selective breeding programs performed in one environment fail to improve the yield of crops grown in another environment (Barrero Farfan et al., 2013). In these and many other examples, such as the difficulty of transferring polygenic scores in human GWAS (e.g. Rosenberg et al., 2019), the context matters enough that it changes the answer in entirely practical ways.

We also know that biotic interactions can be key. For instance, microorganisms often require the presence of their ecological partners in order to grow, a suggestion that was made at the dawn of microbiology (Winogradsky, 1935). This is true even for low-diversity enrichment communities formed in minimal media, where the metabolic activities of all co-occurring species dramatically transform the shared environment, thus permitting the growth of taxa that would otherwise be excluded (Estrela et al., 2020; Goldford et al., 2018). Studies have also found that our ability to isolate and cultivate soil bacteria is dramatically enhanced by embedding culture chambers in soil, further emphasizing the challenges that exist when we separate an individual species from its biotic context (Chaudhary et al., 2019; Kaeberlein et al., 2002; Nichols et al., 2010). Of course, the biotic context impacts not only survival. There is growing evidence, for example, that many human pathogens only become virulent when in the company of other community members (Hajishengallis and Lamont, 2016; Wiles and Guillemin, 2019). In a similar vein, mycorrhizal fungi rely on associated bacteria in order to provide services to their host plants (Minerdi et al., 2008). In all these examples, species studied in isolation would fail to display the defining functional phenotypes of interest.

Further, one might think that a functional and eco-evolutionary understanding of species interactions could be gleaned from a study of pairwise interactions between two species, such as a host and a pathogen. But, yet again, careful dissection of these interactions in *Arabidopsis thaliana* has revealed that these host-pathogen dynamics are driven by a diffuse web of many interactions, including co-occurring host species sharing interacting pathogen species (Karasov et al., 2014). The functional repercussions of this can be profound; for example, an investigation of the evolution of immunity has revealed that humans who live in regions of high pathogen diversity carry alleles with a broader efficacy, as has also been predicted by theory (Manczinger et al., 2019; Nourmohammad et al., 2016). In multispecies microbial consortia, important community functions are often the result of higher-than-pairwise functional interactions (Sanchez-Gorostiaga et al., 2019), and the evolution of species traits is tightly constrained by the network of interactions with co-evolving species (Lawrence et al., 2012; Scheuerl et al., 2020). Lastly, we can see that drivers of species interactions can be found at even higher levels of complexity. For example, Parker et al., 2015 found that disease pressure on host species of a grassland community were predicted not only by the abundance of the one host species of interest, a phenomenon that is predictable by two-species models, but also by the phylogenetic diversity of the entire plant community. The importance of community context in this last example is clear and compelling.

These few examples reveal the perils of ignoring ecological and environmental context. The same is true when we ignore evolutionary change, especially when it occurs on the same timescale as the functional and ecological dynamics in which we are interested. Of course, this has been long recognized in the burgeoning field of eco-evolutionary dynamics (Becks et al., 2012; Hairston et al., 2005; Pelletier et al., 2009; Post and Palkovacs, 2009). Here, we emphasize that these dynamics impinge not only on questions of ecology and evolution but also on biological function, as illustrated below (for more examples, see Box 1).

Box 1. Practical examples of our call to action.

**Reproducibility and predictability of evolution**

Experimental evolution has largely focused on a single species in isolation (from *E. coli* to *Drosophila*), devoid from its eco-evolutionary context (Graves et al., 2017; Herron and Doebeli, 2013; Hsu et al., 2021; Lenski, 2017; Lenski and Travisano, 1994). Fewer experimental evolution studies have been performed in communities (Barraclough, 2015; Barreto et al., 2020; Evans et al., 2020; Fiegna et al., 2015; Lawrence et al., 2012; Scheuerl et al., 2020), and as a result we still know little about how ecological interactions affect parallelism and convergence in evolution. Addressing this void requires experiments in multiple replicates, under conditions with growing levels of contextual richness: i.e. communities co-evolving under well-controlled closed conditions, communities co-evolving when invasive species may also arrive, communities co-evolving within a multilayered trophic chain (e.g. in the presence of predators), communities evolving under natural environmental conditions with realistic population sizes and levels of genetic diversity, etc. These experiments will allow us to address such open questions as: Does historical contingency become more (or less) important as the eco-evolutionary context becomes more complex? Does diversity within and across trophic levels positively (or negatively) affect the reproducibility and predictability of evolution? Are certain emergent ecological properties of communities, such as their collective metabolic functions, their resilience, etc. more predictable as the communities become more complex? If so, how does the emergence of community properties relate to selective pressures? By replicating parallel evolution experiments with growing layers of initial complexity, we will be able to learn at what level of organization (genotype, phenotype, community-level) evolution becomes more (and less) predictable (Lässig et al., 2017), and why.

**Evaluating the robustness and predictability of ecological interactions to eco-evolutionary context**

Are ecological and functional interactions between species in isolation representative of interactions within a more realistic eco-evolutionary context? As communities and environments become more complex, do pairwise interactions between species become more reproducible, or more idiosyncratic? Do pairwise interactions become less relevant in species co-evolution in favor of diffuse interactions? These questions have received attention in different fields, from the microbiome (Bashan et al., 2016), to plant-herbivore systems (Juenger and Bergelson, 1998). In particular, experimental co-evolution between hosts and pathogens is often investigated outside of the context of the complex ecological community where such co-evolution would take place, despite evidence that diffuse co-evolution can be critical (Karasov et al., 2014; Strauss and Irwin, 2004). We call for host-pathogen co-evolution experiments to be carried out with a high-number of replicates and with communities of growing complexity, both at the host and pathogen levels. This will help us understand how exactly the eco-evolutionary context determines pathogenicity (Wiles and Guillemin, 2019), and will allow us to determine whether co-evolution is more or less predictable (and at what level of coarse-graining) than expected from pairwise experiments.

**Reconciling synthetic biology with ecology and evolution**

The importance of eco-evolutionary context in the functioning of engineered or synthetic systems is clear: will synthetic organisms maintain the functions for which they were designed when other species are interacting with them, or as the environment changes? Will they keep their function over evolutionary timescales, which are quite short in cellular populations? Ecological robustness and evolutionary stability should be part of the design of genetic circuits (Cardinale and Arkin, 2012; Tas et al., 2021). Likewise, efforts to ‘edit’ the microbiome in situ should contemplate the ecological and evolutionary response of the community to those genetic changes (Brenner et al., 2009; Chang et al., 2021; Goldman and Brown, 2009; McCarty and Ledesma-Amaro, 2019). In sum, genetically engineered circuits and organisms should be placed in the context where they will be functioning, if we want their engineered functions to be stable over time. Screening circuit function in a growingly complex and varied context (from different single chassis in isolation to combinatorial consortia of growing complexity) can be followed by selection in an iterative manner to identify robust and/or tunable designs that will sustain function at the community-level despite mutations, ecological interactions, or environmental fluctuations (Sánchez et al., 2021).

**Evaluating the robustness of physiological and life-history tradeoffs to eco-evolutionary context**

Physiological constraints and life-history tradeoffs can help us predict organismal behavior and evolution at the phenotypic level (Stearns, 2000; Stearns, 1989), and they can also be leveraged to make predictions about community assembly (Litchman et al., 2015). However, these tradeoffs are often measured for a single species in monoculture under approximately constant environmental conditions (Basan, 2018; Litchman et al., 2007; Scott and Hwa, 2011), and we generally do not know how stable they are in different ecological contexts (Bergelson and Purrington, 1996), nor how evolvable they may be. Measuring these tradeoffs under increasingly complex eco-evolutionary contexts will help us understand their generality. Does increasing the complexity of the ecological context make these tradeoffs less or more robust? Do additional tradeoffs emerge at higher levels of organization (i.e. between functional groups)?

**Understanding regularities in population genomics**

While population genomics generally attempts to infer the action of key evolutionary forces from static patterns that are established on the time scale of 10^5^–10^6^ generations or longer, it is becoming apparent that extremely rapid evolutionary adaptation is commonplace and can be studied in real time. Indeed population genomics has revealed adaptation within tens of generations, sometimes far less, in multiple natural systems such as viruses, *Drosophila*, sticklebacks, mice, Arabidopsis, and even humans (Barrett, 2010; Barrett et al., 2019; Bergland et al., 2014; Exposito-Alonso et al., 2019; Frachon et al., 2017; Hamid et al., 2021; Williams and Pennings, 2020). These studies raise a number of key questions. For instance, if adaptation is generally this rapid, does it also mean that it is very local and thus idiosyncratically sensitive to the exact local conditions? In other words, do all microscopic details matter? And if yes, then how does the averaging of all of these local and variable selective pressures lead to the signatures of selection established over longer periods of time? In other words, can we reconcile rapid evolution on ecological timescales with evolution on longer timescales? Moreover, is rapid adaptation just a ‘phase’ of adaptation on longer timescales, such as between species, or is it an entirely different process governed by different dynamics, involving different phenotypes, and distinct genetic architecture? Beyond linking evolutionary dynamics across time scales, the elucidation of the genomic architecture of adaptation, and identification of specific loci of adaptation, we believe it is also important to search for interpretable regularities of evolution across species and genomes. For instance, do the same genes underlie adaptation across species at different time scales (Stern and Orgogozo, 2008)? Does the redundancy in genetic encoding of a phenotype mean that the reliable coarse-graining must take place at the level of phenotype and not genotype? Or maybe, despite all of this redundancy, only some loci are likely to undergird adaptation, at least at some key time scales. Finally, while much of the genomic analysis of function tends to focus on the conserved genomic regions, the standing genetic variation that drives rapid evolutionary changes might present exceptionally interesting variants for understanding of organismal function. A common thread in the examples described above is that they are predicated on our ability to find the right level of coarse graining. We seek to understand to what extent increasing the contextual richness and realism will make functional biology more predictable, rather than less.

One recent, compelling example revealed that negative frequency-dependent competition amongst strains of malaria, and the selection that follows from this competition, establish a pattern of limiting similarity that makes the community more robust to perturbations (He et al., 2021). This demonstrates how ecological and evolutionary processes jointly determine community resilience, an important aspect of community function. In this example, the applied relevance is clear - interventions to control malaria must be more prolonged than currently implemented.

The relevance of eco-evolutionary dynamics is especially clear when we consider cancer, which we now understand to be a process of the evolution of competing somatic lineages. Cancer dynamics depend in crucial ways on the ecological dynamics of the complex cellular communities inside the body, involving interactions and cross signaling among multiple dividing, dying and differentiating cell types such as stroma, tumor, and immune cells (Rodrigues et al., 2021). The current interest in studying cancer within the richer biological context of organoids and genetically modified mouse models reflects the realization that cancer is unlikely to be fully understood by studying cell lines in test tubes, instead requiring consideration of the community of interacting and coevolving cells and cellular lineages. We also know now that even targeted cancer therapies impact tumor growth by affecting not just the tumor cells but also the immune system (Deng et al., 2018; Goel et al., 2017). The same is true for the cancer therapies aimed at the immune system that work very differently depending on the genotype of the tumor (Song et al., 2020).

Similarly, the etiology of many viral diseases is at its core an eco-evolutionary process that connects evolutionary dynamics of large viral populations to the shifting ecological landscape of the immune system. The appropriate scale may be within the body (e.g. HIV), or across population(s), and in relation to dynamics that may involve communities of interacting species (e.g. dengue, flu, SARS-CoV-2; Gibb et al., 2020; Gruber, 2017). The ability of HIV to evolve sufficiently quickly to outrun the immune system and maintain resistance is not the context of function - it *is* function, at least in the sense that this is what causes the disease of immune deficiency. Along these same lines, the ability of viruses to jump from one species to another, evolve an ability to infect, and then spread, is currently responsible for shutting down the economies of the whole world. Again, evolution is the ultimate cause of these problems, not just some extra context and nuance.

## Is it hopeless?

Given this vast complexity, and accepting that it often matters in key ways, how are we to make progress? If it is not effective to simplify our systems at the outset, must we give up the aspiration for simplicity of explanation? As we argue below, not necessarily.

First, complexity at one level of organization in no way precludes simplicity of explanation at a higher level. In fact, microscopically, *all* biological systems are impossibly complex. Predictable properties can only arise at some higher levels of organization: that is, simplicity is *always* emergent. Even something as ‘trivial’ as the exponential growth law of a population summarizes a tremendous complexity of microscopic processes of growth and reproduction. The exponential growth is not a property of any member of the population; it is a property of the entire population. Similarly, the enzymatic kinetics of Michaelis and Menten are immeasurably simpler than the microscopic laws of quantum chemistry ‘under the hood’, and our description of cell physiology rarely invokes the latter. At the next level of organization, the simplicity of quantitative phenomenological growth laws (Schaechter et al., 1958) has been ‘a source of wonder and inspiration’ (Scott and Hwa, 2011). Despite the immense underlying complexity of genetic and metabolic regulation, growth laws can be largely explained by a simple, coarse-grained argument of proteome allocation into ribosomal vs. non-ribosomal components (Basan et al., 2015; Scott et al., 2010). At higher levels of organization, organismal traits may be predicted from the laws of mendelian genetics, which were originally derived without requiring any knowledge of the molecular or cellular processes from which they emerge and well before we even knew that DNA was the molecular carrier of genetic information. No physiological or molecular knowledge is required either to calculate genotype frequencies under Hardy-Weinberg equilibrium, or the effects of drift and selection on allele frequency. Importantly, these and other fundamental results of population genetics describe emergent population-level properties, which lack meaning at the level of a single individual organism. Throughout the history of biology, our successes were never about simplifying away the complexity of a system, but about finding the appropriate coarse-graining.

Can simplicity of functional understanding arise also in systems of interacting and co-evolving populations and species? We suggest yes, partly based on conceptual argument and partly as a matter of empirical evidence. Consider the assembly of microbial communities, which typically consist of hundreds or thousands of taxa. At first glance, it would seem impossibly complex. And yet, recent studies illustrate that microbial ecosystems represent an illuminating example of predictable coarse-grained organization. For instance, the microbiomes of different individual bromeliad plants contain highly similar fractions of their metagenomes devoted to respiration, fermentation, methanogenesis, and other metabolic functions despite varying widely in the species they contain (Louca et al., 2017; Louca et al., 2016). A logical conclusion from this observation is that predictable, species-level compositions are not required for generating predictable dynamics at the level of metabolites. Such emergent simplicity arises generically in consumer-resource models (Goldford et al., 2018; Marsland et al., 2019), and it is also exhibited by enrichment communities assembled in simple synthetic environments, where ecological sources of variation (e.g. migration, abiotic factors, bottlenecks, etc.) can be controlled (Estrela et al., 2020; Goldford et al., 2018). In minimal sugar-limited environments, for example, two dominant metabolic groups are consistently found: One group is made by respiro-fermentative bacteria, which metabolize the lion's share of the sugar, while the second group is made by respirative bacteria, which specialize on the organic acids that are secreted as overflow metabolic byproducts by the former. The ratios of cell counts in each of these two metabolic groups are quantitatively consistent across communities that were started from different species pools in this system (Estrela et al., 2020; Goldford et al., 2018; Marsland et al., 2019). A similar metabolic organization has been found to repeatedly arise in experimental evolution studies that started from a single isogenic strain (Good et al., 2017; Rozen and Lenski, 2000; Treves et al., 1998). For instance, Rosenzweig and colleagues (Kinnersley et al., 2009; Rosenzweig et al., 1994) found that after only 700 generations in a glucose-limited chemostat, an initially isogenic population of *E. coli* had diversified into an ecosystem composed of four derived strains. One strain had adapted by increasing the rate of glucose uptake, while the others had specialized in the increased overflow metabolic secretions of the glucose specialist.

We highlight this example because it demonstrates elegantly that both top-down selection among a diverse pool of taxa and bottom-up community assembly via evolution from a single genotype lead to communities that predictably assemble into convergent functional structures. This reproducibility can be explained from a metabolic tradeoff that is fundamentally conserved across all kingdoms of life, where fast-growth in sugars is associated with the secretion of overflow metabolites into the environment. The amount of these byproducts produced per unit glucose consumed is governed by the glucose uptake rate: the faster a bacterium metabolizes glucose, the larger the amount of byproducts it releases (Basan et al., 2015). This leads to a tight covariation between the magnitude of both, even for isolates that belong to different genera (Estrela et al., 2020). Genome-scale metabolic models can correctly estimate the ratio of both metabolic groups by making such simple assumptions.

In these examples, predictability was enabled by the fact that metabolism is highly structured, only some solutions are sensible or mathematically possible, and bacterial taxa have been coevolving with each other for eons, making microbial communities very nonrandom. In a similar way, biochemical, physiological, and physical constraints, combined with (co)evolution, might often lead to predictable and, hence, potentially understandable outcomes in other microbial and non-microbial systems. It is our contention and hope that co-adaptation and co-evolution, especially when it comes to functionally important community or individual traits, will be governed by a limited number of driving forces that, when revealed, will allow for a simpler coarse-grained understanding to emerge.

## Path forward

So what are we proposing? As mentioned at the outset, we do not aim to rehash previous discussions on holism vs reductionism. This is not the conversation we are trying to have: the limitations of a reductionist approach are well established, as is the fact that the tremendous progress the biological sciences have experienced over the past centuries, both fundamental and applied, has been due almost entirely to this very approach. Our aim is not to argue that attempts to reconstruct the whole from pieces is futile. Neither are we asking for more and more complexity for complexity’s sake. Instead, we argue two points.

First, we must remember that ‘what the pieces do’ may not be clear when the whole is extracted from its biological context. This, of course, is not a new insight - we all know it to be true. When we design our experiments around simplified environments, it is not out of a belief that the natural context doesn't matter, but as a matter of feasibility. Our point here is that the time is ripe to act upon this knowledge, and eliminate the barriers of technological convenience. This is essential because the tools that we use shape the research that we do, and as we get better and better sequencing, for example, the comparative lack of tools for high-throughput phenotyping is becoming increasingly a problem. The danger is to keep zooming in on the increasingly microscopic details of species in isolation, where the existing street lights shine brightest. We therefore call for redoubling our efforts to develop new technology for high-throughput functional characterization of organisms in natural or semi-natural habitats of growing eco-evolutionary context (Mallard et al., 2020).

Second, if we are to find the right level of coarse graining, it is imperative to pick model systems of ‘appropriate’ contextual richness and with sufficient replication. But how much is enough? At the outset, we cannot know. Thus, we're advocating for model systems that can be integrated into an axis of tunable contextual richness: lab, semi-natural, and natural. As we increase the size and complexity of experimental systems in a controlled manner, we will search for emergent properties that only reveal themselves at higher levels of organization (and at the appropriate coarse-graining). We can attempt to search for these emergent properties in a systematic manner, rather than discovering them by serendipity. Such a concerted effort will benefit from looking across a diverse array of organisms because only then will consistent, and likely robust, emergent patterns become recognizable. This experimental methodology would go hand in hand with the recent calls in theoretical literature for models of ‘appropriate complexity’ (Getz et al., 2018). To make our proposal more concrete, we present a few representative areas in functional evolutionary biology (Box) where such an approach may be applied and plausibly push the field forward.

Finding the appropriate level of coarse-graining remains a matter of art, an art that becomes harder and harder as the systems become more complex. One exciting possibility is that machine learning and AI methods for performing automated variable selection for learning sparse predictive models will prove effective. However, there are no easy solutions here. Just like more data are not yet progress, a bigger computer alone will not suffice: AI can discover phenomenological regularities, but for such regularities to clue us into underlying causal forces and guide our interrogation of biological systems, data-intensive approaches need to be integrated with the eco-evolutionary theory of functional biology. These approaches will also inform the development of experimental systems in ecology and evolution that capture the appropriate eco-evolutionary context for organismal function, at least to some phenomenological degree.

Many of the most pressing problems facing humanity - from global change, to infectious disease and zoonosis, to cancer - are all problems of eco-evolutionary function. Confronting these problems will require that we build a sufficient understanding that we can predict, and even control, how these systems function as they evolve. These are challenging and yet crucial problems that will require concerted collaboration across currently disparate fields that extend much beyond the traditional boundaries of Ecology and Evolution, and include all branches of Organismal and Functional Biology, as well as Physics, Engineering, Statistics, and Computer Science.

As we have argued, the enterprise of studying organisms in isolation and as static, already evolved entities, while hugely successful, is also profoundly limited. Our belief, which lies in contrast to Jacques Monod’s famous dictum — ‘Anything found to be true of *E. coli* must also be true of elephants’ — is that much of what one learns about the functional behavior of *E.coli* in isolation hardly even extends to *E.coli* in the gut, let alone to elephants in the savannah. The heart of our proposal, here, is thus the creation of a new field of **Function of Evolving Systems** that focuses on the function of organisms in the communities in which they reside, over periods of time when interactions evolve. It is both exciting intellectually, and essential practically.
